# Targeting circulating tumor cells to prevent metastases

**DOI:** 10.1007/s13577-023-00992-6

**Published:** 2023-10-24

**Authors:** Karol Gostomczyk, Mohammed Dheyaa Marsool Marsool, Hamnah Tayyab, Anju Pandey, Jędrzej Borowczak, Facundo Macome, Jose Chacon, Tirth Dave, Mateusz Maniewski, Łukasz Szylberg

**Affiliations:** 1 Department of Obstetrics, Gynaecology and Oncology, Chair of Pathomorphology and Clinical Placentology, Collegium Medicum in Bydgoszcz, Nicolaus Copernicus University, Torun, Poland; 2 University Hospital No. 2 Im. Dr Jan Biziel, Ujejskiego 75, 85-168 Bydgoszcz, Poland; 3 Department of Tumor Pathology and Pathomorphology, Oncology Centre, Prof. Franciszek Łukaszczyk Memorial Hospital, Bydgoszcz, Poland; 4 University of Baghdad, Al-Kindy College of Medicine, Baghdad, Iraq; 5 Mayo Hospital, Lahore, Pakistan; 6 Kasturba Medical College, Manipal, India; 7 Universidad del Norte Santo Tomás de Aquino, San Miquel de Tucuman, Argentina; 8 American University of Integrative Sciences, Cole Bay, Saint Martin, Barbados; 9 Bukovinian State Medical University, Chernivtsi, Ukraine; 10 Chair of Pathology, Dr Jan Biziel Memorial University Hospital No. 2, Bydgoszcz, Poland

**Keywords:** CTCs, TAMs, Targeted therapies, Detection, Metastases, Cancer

## Abstract

Circulating tumor cells (CTCs) are cancer cells that detach from the primary tumor, enter the bloodstream or body fluids, and spread to other body parts, leading to metastasis. Their presence and characteristics have been linked to cancer progression and poor prognosis in different types of cancer. Analyzing CTCs can offer valuable information about tumors’ genetic and molecular diversity, which is crucial for personalized therapy. Epithelial-mesenchymal transition (EMT) and the reverse process, mesenchymal-epithelial transition (MET), play a significant role in generating and disseminating CTCs. Certain proteins, such as EpCAM, vimentin, CD44, and TGM2, are vital in regulating EMT and MET and could be potential targets for therapies to prevent metastasis and serve as detection markers. Several devices, methods, and protocols have been developed for detecting CTCs with various applications. CTCs interact with different components of the tumor microenvironment. The interactions between CTCs and tumor-associated macrophages promote local inflammation and allow the cancer cells to evade the immune system, facilitating their attachment and invasion of distant metastatic sites. Consequently, targeting and eliminating CTCs hold promise in preventing metastasis and improving patient outcomes. Various approaches are being explored to reduce the volume of CTCs. By investigating and discussing targeted therapies, new insights can be gained into their potential effectiveness in inhibiting the spread of CTCs and thereby reducing metastasis. The development of such treatments offers great potential for enhancing patient outcomes and halting disease progression.

## Introduction

Circulating tumor cells (CTCs) are cancer cells that detach from a primary tumor site and enter the bloodstream or body fluids. They are considered promising cancer biomarkers by the American Society of Clinical Oncology (ASCO) [1], and their presence and distinct characteristics are associated with progression and poor prognosis in various cancers [2]. Due to shear stress, immune surveillance, and unfavorable microenvironment, most CTCs last in the circulation only for a brief time. As such, their number in patients’ blood does not typically exceed ten cells per milliliter of blood [3]. CTCs interact with components of the tumor microenvironment, such as tumor-associated macrophages (TAMs) and fibroblasts, and promote cancer spread to distant organs. Therefore, targeting and eliminating CTCs can potentially prevent metastasis and improve patient outcomes.

The detection of CTCs is an area of active research. Several techniques, including immunocytochemistry, fluorescence in situ hybridization (FISH), microfluidics, and next-generation sequencing, have been developed to identify CTCs in diagnostic samples [4]. Monitoring CTCs levels during treatment can help assess treatment response and identify patients at higher risk of metastasis [4, 5]. The analysis of CTCs provides insights into tumors’ genetic and molecular heterogeneity, guiding personalized therapy. New strategies targeting CTCs, such as immunotherapy, targeted therapies, and nanotechnology-based approaches, are currently being explored to prevent cancer spread [4].

CTCs play a vital role in the development of tumor immune modulation. Numerous interactions with the tumor immune microenvironment increase their potential to metastasize and escape host immune surveillance [5]. TAMs, transglutaminase 2 (TGM2), a cluster of differentiation 44 (CD44), Epithelial cell adhesion molecule (EpCAM), and vimentin promote tumor growth and metastasis by supporting the infiltration and activity of effective immune cells and facilitating CTCs extravasation at the site of metastasis. This review presents recent progress in detecting CTCs, the mechanisms of their interactions, and the development of therapies targeting CTCs to prevent cancer metastasis. We aim to summarize the recent reports to improve the general understanding of CTCs interactions and their role in treatment selection. We will also structure the new insights to facilitate further clinical trials.

## Methods and selection criteria

### Methods

We searched multiple medical databases, including PubMed and Google Scholar, and identified articles containing selected keywords. The search terms included “circulating tumor cells,” “CTCs,” “tumor-associated macrophages,” “TAMs,” “TGM2,” “CD44,” “EpCAM,” “vimentin,” “metastases,” “flow cytometry in cancer,” “macrophages,” “circulating tumor cells therapies,” “tumor-associated macrophages therapies,” “epithelial-derived cancer,” “mesenchymal-derived cancer,” “glioblastoma,” “melanoma,” “CTCs detection.”

### Inclusion criteria

Research articles were selected and assessed only if they met the following criteria

1. Cancer-related research.
2. Reporting of cancer prognosis and metastases.
3. Reporting of cancer therapies targeting CTCs and TAMs.
4. Studies published in English with indicated dates and locations and indexed in MEDLINE.
5. Studies that include cancer detection, therapies, and perspectives.
6. Studies that address the role of CD44, TGM2, EpCAM, and vimentin in cancer detection and perspectives.
7. Studies that address the role of CD44 and TGM2 in cancer mechanisms and interactions.
8. Studies that address the role of CTCs and TAMs in metastases and cancer prognosis.

### Exclusion criteria

Letters to the editor, abstracts without full text, and studies related to some extent to the content of the article but containing outdated data were not included in this review.

## Circulating tumor cells: overview

### Epithelial-mesenchymal transition and mesenchymal-epithelial transition

**Fig. 1:**
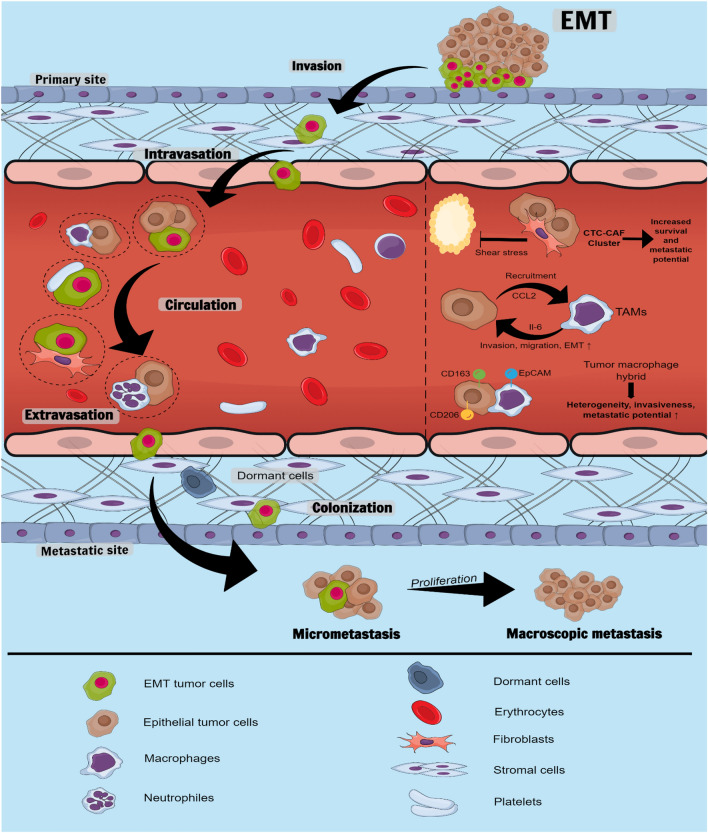
The CTCs interactions’ impact on metastasis. Active intravasation requires CTCs to undergo EMT at the primary site. Acquiring mesenchymal properties enables CTCs to escape immune surveillance and survive in circulation. Platelet coating and interactions with CAFs, TAMs, and WBCs hinder antigen presentation, protect CTCs from shear force, and increase their metastatic potential. TAMs and CAFs form clusters with CTCs—those heterogeneous structures consist of broad populations of epithelial and mesenchymal CTCs that are more invasive and likely to arrive at the target pre-metastatic niche. This complicated crosstalk favors CTCs’ survival and migration and facilitates the formation of distant metastasis. *EMT* epithelial-mesenchymal transition, *CCL2* chemokine C–C motif chemokine ligand 2, *CD* a cluster of differentiation, *EpCAM* Epithelial cell adhesion molecule, *IL* interleukin, *TAMs* tumor-associated macrophages, *CTC-CAF* cluster—circulating tumor cell-cancer-associated fibroblast cluster

Several routes of CTCs infiltration into the bloodstream and body fluids have been described. Passive infiltration is known as the non-epithelial-mesenchymal transition (non-EMT). This mechanism is initiated by external mechanical forces, which force epithelial cells into circulation [6]. Since non-EMT CTCs are scarce and almost immediately eliminated by circulating immune cells, their role in metastasis is negligible. During active infiltration, epithelial CTCs acquire mesenchymal-like features, which allow them to gain mobility, detach from the primary tumor site, and enter the bloodstream. This phenomenon, called epithelial-mesenchymal transition (EMT), is critical for cancer progression. Suppose those cells survive in the circulation and arrive at the metastatic site. In that case, they undergo a reverse process called mesenchymal-epithelial transition (MET), which allows them to bypass the vascular endothelium and form micrometastases (Fig. 1) [7].

EMT is a multidimensional process initiated by the interactions between tumor cells in their microenvironment. Epithelial CTCs have closely adjoined and polarized structures maintained through cell–cell adhesion and junctions. There are five cell junctions: tight junctions, adherens junctions, desmosomes, hemidesmosomes, and gap junctions [8]. All of them are necessary to maintain the integrity of epithelial tissues. In contrast, mesenchymal cells do not exhibit adhesion or polarity; thus, they are mobile and can bypass the epithelial barrier [7]. The switch from epithelial to mesenchymal phenotype requires the downregulation of epithelial markers, such as E-cadherin, and the upregulation of mesenchymal markers, including N-cadherin, fibronectin, vitronectin, and smooth-muscle actin [7, 8]. During MET, this process is reversed—mesenchymal cells transform into epithelial cells, epithelial markers undergo upregulation, and mesenchymal markers undergo downregulation [7].

### Application in research

CTCs have numerous applications in cancer research and treatment. The CellSearch system is currently considered the gold standard for CTCs detection [9]. It detects peripheral CTCs and provides information regarding the tumor and the patient’s prognosis in conjunction with clinical outcomes. CTCs can monitor residual disease, assess treatment response, and track disease progression and tumor evolution, including developing resistance to therapy [10]. Their distinctive features are associated with patients’ overall survival and progression-free survival [2].

In early breast cancer (EBC), disseminated tumor cells (DTCs) in the bone marrow were associated with a worse prognosis. Braun et al. analyzed 4,703 EBC patients and found that those with DTCs had worse clinical and laboratory outcomes before treatment [11]. Janni et al. analyzed data from 3,173 EBC patients from five breast cancer institutes. They found that the presence of CTCs was associated with larger tumors, increased lymph node involvement, higher-grade tumors, and lobular tumor type [12]. The authors found no significant association between the presence of CTCs, hormone-receptor status, or HER2 status. CTCs were an independent prognostic factor for shorter, disease-free, breast cancer-specific, and overall survival. CTCs were not significantly associated with prognosis in patients without lymph node involvement or triple-negative breast cancer. Since, unlike in metastatic breast cancer, there is no established cutoff value for CTCs in EBC, the presence of CTCs may facilitate prognosis prediction [13].

Personalized treatment of metastatic lung cancers relies heavily on biomarker testing, especially oncogene-addicted cancers, which can be treated with tyrosine kinase inhibitors (TKIs) [14]. While CellSearch is an FDA-approved system for monitoring breast and colon cancers, it has yet to be validated for lung cancer. Consequently, circulating tumor DNA (ctDNA) is favored over CTCs in the diagnosis and outcome prediction of lung cancer patients [15]. Recently, Krebs et al. compared two methods for detecting CTCs in advanced lung cancer patients: a surface marker-dependent method using CellSearch for EpCAM + cells and a surface marker-independent method based on isolation by the size of epithelial tumor cells (ISET) [16]. Out of 40 patients, only 23% had CTCs detected using the surface-marker approach, while the ISET tests detected CTCs in 83% of cases. However, we found no studies comparing the applicability of ctDNA versus CTCs in detecting and monitoring lung cancer.

### Advantage over the biopsy

CTCs offer several advantages over traditional biopsy methods [17]. CTCs can be obtained non-invasively, allowing for repeated sampling without invasive procedures and reducing the number of adverse events compared to conventional biopsies [18–20]. CTCs acquisition is more accessible than accessing target tissue via traditional biopsies; therefore, they provide an alternative method of molecular genotyping, especially when the cancer’s primary site is unknown or when the quantity of tissue available for analysis is limited [21]. CTCs’ phenotype and dynamics change over time and reflect cancer progression; hence, their rapid testing provides real-time prognostic data, guiding personalized therapy [21, 22]. CTCs can be rapidly tested, providing real-time information about tumor progression. Furthermore, CTCs can help reduce diagnosis bias from tumor heterogeneity, providing a more comprehensive understanding of the tumor’s biology [23, 24].

### Diversity among clusters

CTCs exhibit extreme phenotypic heterogeneity, existing as individual cells or clusters of 2 to 50 cells. They form through collective invasion, passive shedding, and aggregation of individual tumor cells during migration and in circulation [25]. Their appearance predicts the switch toward a mesenchymal-like phenotype [25, 26]. However, the lack of a comprehensive analysis of CTCs heterogeneity at genetic, phenotypic, and morphological levels is a significant challenge in characterizing their impact on metastatic progression [26].

The metastatic potential of CTCs depends on the epigenetic modifications of their signaling. Genes associated with cell stemness and proliferation responsible for their higher metastatic potential are usually hypomethylated and, therefore, more active in cluster CTCs. Genomic changes can give rise to different subclones within the tumor and CTCs, and the subsequent metastasis process is guided, at least in part, by epigenetic reprogramming [26].

### Interactions among clusters and metastases

CTCs clusters are enriched by adhesive proteins, such as plakoglobin, CD44, or claudin-11 [25]. They respond to alterations in the extracellular matrix, remodeling and shaping the structure of intercellular junctions within the tumor microenvironment [27]. Increased intercellular adhesion allows the clusters to intravasate and maintain stem-like properties necessary to form micrometastasis in distant organs. The knockdown of pro-adhesive signaling abrogates CTCs cluster formation and suppresses metastasis [25].

CTCs interact with blood cells and use them to enhance their adhesive properties and facilitate metastasis [25]. CTC-neutrophil clusters found the blood of women with advanced-stage breast cancer was associated with a higher risk of early metastasis [28]. After injecting CTCs from CTC-neutrophil clusters into the bloodstream of tumor-free mice, Szczerba et al. observed substantially increased metastases. In contrast, eradicating neutrophils in mice with breast tumors delayed cancer spread to the lungs [28].

In the circulation, platelets rapidly coat CTCs and facilitate cluster formation by interacting with adhesive proteins, such as fibronectin and integrins. Platelet-coated CTCs are more likely to escape immune surveillance due to impaired antigen presentation [29]. At the same time, the MHC I complexes transferred to CTCs from platelets give them a new self-identity that prevents NK cell-mediated cytolytic attacks. Platelets also inactivate natural killer (NK) group 2D receptors on NK cells and T lymphocytes, protecting CTCs against immune response [30]. Despite this support, CTCs survive in circulation only for a short time (1–2.4 h) due to shear stresses and apoptosis induced upon losing the attachment to neighboring cells or extracellular matrix [30].

The literature suggests the existence of other types of CTC, in which detection and analysis are far more challenging. For instance, lymphatic circulating tumor cells (L-CTCs) are commonly found in lymphatic vessels, which are tiny, colorless structures characterized by low intraluminal pressure and low cell concentration; thus, their visualization requires additional labeling and mapping using lymphography [31]. Lymph sampling is tedious and methodologically challenging and rarely performed in clinical practice. Since acquiring even a few milliliters of lymph for conventional in-vitro assays (such as flow cytometry, PCR, and genomic/proteomic tests) may be difficult, the interactions and metastatic potential of L-CTCs still need to be established [31].

The mechanical pressure and permission blood flow are key factors driving CTCs extravasation and subsequent metastatic growth [32]. Regions with low hemodynamic flow are most suitable for CTCs to engage with endothelial cells. When the adhesive capacity of CTCs surpasses the shear forces of the blood flow, they attach to the walls of blood vessels at distant sites. There, single CTCs can sequentially form intravascular clusters [32]. Once fixed in the microvasculature, they generate immune-interacting molecules that strengthen the adhesion to endothelial cells and enable CTCs’ extravasation (Fig. 1) [32].

## Circulating tumor cells detection

### Molecular markers

EMT and MET are fundamental for CTCs to acquire mobility, resistance to apoptosis, and intravasate and metastasize [33]. During both processes, cells possessing epithelial-mesenchymal properties switch their phenotypes to adapt better to the local microenvironment [34]. These changes are associated with altered expression of specific proteins, which may become potential therapeutic targets. Some CTCs markers indicate the primary tumor derivative. In contrast, others, such as EpCAM and vimentin, are present in every tumor cell and can be potentially used to detect every type of CTCs [35, 36].

Interestingly, De Wit et al. used filtration and fluorescent labeling to detect EpCAM-negative CTCs in the blood of patients with metastatic lung cancer. The presence of EpCAM-negative CTCs doubled the number of CTCs and CTC-positive patients in this cohort, but EpCAM-negative CTCs were not associated with the patient’s prognosis [37]. DeWit et al.’s results indicate that EpCAM may not be suitable for detecting mesenchymal-like CTCs, which lose the expression of EpCAM during EMT, invalidating the theorem of “universal” CTCs markers [38].

Epithelial cell adhesion molecule (EpCAM) is expressed only by epithelial cells and partakes in all steps of the metastatic cascade. Its expression decreases during EMT but can still be detected on every CTC, regardless of its epithelial-mesenchymal phenotype [35]. EpCAM mediates the adhesion of tumor cells to its primary site. Therefore, its loss is crucial for increasing the migratory potential of cancer cells. EpCAM enables binding between adjacent CTCs in clusters by forming tight and adherens junctions [35, 39]. EpCAM also facilitates CTCs adhesion to distant metastatic sites [39].

Vimentin is an intermediate filament protein that stabilizes the intracellular structure. Its high expression is typically associated with mesenchymal cells and is upregulated during EMT [36]. It aids CTCs in dissociating from the primary site and acquiring invasive properties. Vimentin facilitates the adaptation to the new microenvironment, forming metastatic attachment and promoting colonization of metastatic sites [36].

CD44 and TGM2 are other critical mediators of EMT and MET [40, 41]. CD44 is a cell surface glycoprotein that regulates cell interactions and microenvironment interactions. Its upregulation has been associated with cancer progression, invasion, and metastasis [42]. TGM2 is a multifunctional enzyme that promotes cell adhesion, migration, and extracellular matrix remodeling [40]. CD44 and TGM2 are present in every CTCs and gained attention as novel detection markers [41, 43] and potential therapeutic targets [40]–[42, 44].

While the links between CD44, TGM2, and metastasis have already been established, further research is needed to advance our understanding of the role of CD44 and TGM2 in EMT and MET [41, 43, 45]. It is also essential to evaluate their potential as detection markers and consider their implementation in CTCs-targeting therapies to prevent metastasis.

### Detection technologies

Transitioning from CTCs detection in a laboratory to clinical practice remains challenging [46]. Only the CellSearch system has been approved by the FDA to detect CTCs (Table 1), but it has some limitations [47]. CellSearch employs antibody-coated magnetic beads to isolate and enrich captured cells based on EpCAM expression. It uses in-vivo enrichment and ligand capture to ensure high CTCs purity [46]. Capturing ligands with density gradient sedimentation may cause interference with free microbeads.

**Table 1:** CTCs detection methods in cancers

Device name	Cancer	Study results and specifications
CELLSEARCH® (Janssen Diagnostics) [48, 49]	BRCA, PD, OVCA, CRC [48, 49]	Low purity of captured CTCs; variable sensitivity (20–70%); variable specificity (89% to 99.7%) [48, 49]
AdnaTest (Adnagen) [50]	BRCA, PCa, OVCA, CRC [50]	Blood and bone marrow samples analysis; downstream RNA analysis after RT—PCR enrichment; high sensitivity (73%); frequent WBCs contamination; detection limit ≥ 2 CTCs per 7.5 mL sample [50]
MACS system (Miltenyi Biotec) [51]	NSCLC, BRCA [51]	The detection of EpCAM-negative, CK-positive CTCs; can be used with leukocyte depletion, achieved through negative enrichment via anti-CD45 antibodies [51]
MagSweeper (Illumina) [52]	BRCA, PCa, CRC [52]	High sensitivity (100%); high purity of captured CTCs (~ 100%); high throughput processing of 9 mL/hr; able to detect 1—3 CTCs/mL [52]
GILUPI CellCollector™ [53]	BRCA [53]	Invasive and time-consuming method; in-vivo-based detection; may soon process large volumes of blood [53]
Modular Sinusoidal Microsystems (BioFluidica) [48]	PDAC [48]	Impedance-sensing cell enumeration; determination of cell viability; high yield and purity of captured CTCs (> 86%); processing up to 7.5 mL of blood per hour [48]
Herringbone (HB) Chip [54]	PCa [54]	Processing up to 4.8 mL/h; detection limited to 12 CTCs/ mL; low purity of captured CTCs (~ 14%); a limited number of conducted preclinical studies [54]
GEDI [55]	BRCA, PCa [55]	High capture specificity and sensitivity (94%); high purity of captured CTCs; can detect up to 27 CTCs/mL [55]
GEM Chip [56]	PDAC [56]	Antibodies-based method; it possesses high selection efficiency; processes 3.6 mL/h [56]
OncoCEE (Biocept) [57]	BRCA [57]	Feasible for the analysis of CK + and CK- CTCs; high probability of capturing CTCs; 95% sensitivity and 92% specificity [57]
LiquidBiopsy® (Cynvenio) [58]	BRCA, LC [58]	Processing of 5 mL/h; high purity of detected cells; the sheath flow reduces non-specific binding; error accuracy of 20% and error precision of 25% [58]
Graphene oxide (GO) Chip [59]	BRCA, PDAC, LC [59]	Processing of 1–3 mL/h with high capture yield; high but variable sensitivity (73% ± 32.4% at 3–5 cells per mL of blood); limited clinical validation [59]
Ephesia (CTC-Chip) [60]	BRCA, NSCLC, PCa, CRC [60]	High capture specificity; the processing of more than 3 mL/h; it maintains the viability of 98% of captured cells; high sensitivity (99.1%) and specificity (100%) [60]
IsoFlux (Fluxion) [61]	BRCA, PCa [61]	It detects genetic alterations with a CTCs capture rate of 50% [61]
Quadruple magnetic separator [61]	BRCA [61]	Minimal preclinical data; detects heterogeneity among CTCs through immunofluorescence; requires multiparameter analysis [61]
CTC-iChip [62]	EpCAM-positive cancer, EpCAM-negative cancer [62]	Under development by Janssen Diagnostics, it utilizes positive and negative enrichment and combines size-based separation of WBCs; processing of 8 mL/h; detection limit of < 30 CTCs/ 7.5 mL; very low purity of captured CTCs (~ 8%) [62]

*CTCs* circulating tumor cells, *EpCAM* epithelial cell adhesion molecule, *LC* lung cancer, *NSCLC* non-small-cell lung cancer, *BRCA* breast cancer, *PCa* prostate cancer, *PDAC* pancreatic ductal adenocarcinoma, *OVCA* ovarian cancer, *CRC* colorectal cancer, < *WBCs* white blood cells, *CD45* cluster of differentiation 45, *CK* cytokeratin, *RT-PCR* reverse transcription polymerase chain reaction

Furthermore, size exclusion filtration and ligand capture are time-consuming and have low throughput, while barcode particles are yet to be automated. Self-propeller micromachines have uncontrollable motion direction and velocity, and magnetic beads cannot capture CTCs with low expression of biomarkers. Moreover, microfluidic chips with ligand capture have a slow flow rate, leading to long CTCs enrichment times.

## Targeting circulating tumor cells

CTCs emerge as a hallmark of disease progression and indicate prompt metastasis [63]. During the epithelial-mesenchymal transition (EMT), cancer cells acquire stem-like properties and detach from the original tissue [64]. In the bloodstream, CTCs activate multiple mechanisms, including platelet clothing and the secretion of growth factors, to evade immune surveillance. If not recognized by immune cells, CTCs will extravasate and form metastasis [65]. CTCs are associated with poor prognosis in hepatocellular carcinoma [66], lung [67, 68], and bladder cancer [69]. Therefore, their early diagnosis and eradication may prolong patients’ survival [70]. Despite the recent progress in detecting CTCs, designing a reliable therapeutic approach seems much more challenging. Therefore, we will summarize current therapies targeting CTCs and review their clinical utility (Table 2).

**Table 2:** Studies targeting CTCs

Study settings	Intervention	Results	Conclusions
An orthotopic HCC model [71]	Primary tumor resection [71]	Decreased CTCs volume; restricted hematogenous dissemination, tumor growth, and early and advanced distant metastases [71]	Early metastases might reflect hematogenous spread, while its accumulative effect might manifest as advanced metastases [71]
HCC patients [73]	Primary tumor resection [73]	Increased CTCs counts after liver resection were associated with a shorter OS and DFS in HCC patients [73]	The postoperative increase in the CTCs count is associated with tumor thrombi and may be reduced by the “no-touch” approach [73]
CRC patients [72]	Primary tumor resection [72]	The clearance of circulating tumor cells after CRC excision [72]	The clearance of CTCs was the greatest in tumors with the best prognosis [72]
NSCLC patients [108]	Primary tumor resection [108]	Decreased CTCs-positivity (51.8% vs. 32.1%), CTCs count (3.16 vs. 0.66 per 10 ml), and EGFR expression (89.7% vs. 38.9%) one month after surgery [108]	Postoperative presence of CTCs was associated with disease recurrence and was an independent prognostic factor of reduced DFS [108]
PDAC patients [77]	Primary tumor resection [77]	CTCs significantly decreased after surgery in chemo-naive and neoadjuvant patients. CTCs count was higher in patients with more advanced diseases [77]	High CTCs pre- and post-surgery predicted disease recurrence. Epithelial, mesothelial, and total CTCs counts were associated with early recurrence [77]
Oligometastatic prostate cancer [109]	Cytoreductive prostatectomy [109]	17/33 CTCs-negative patients before surgery. 21/33 CTCs-negative patients after surgery [109]	≥ 2 CTCs before or six months after surgery predicted a worse clinical outcome. ≥ 5 CTCs predicted shorter OS in metastatic prostate cancer. CTCs might help select patients who benefit from cytoreductive prostatectomy [109]
PDAC patients [110]	Chemotherapy (5-fluorouracil) [110]	Reduced CTCs in blood. Decreased CTCs burden after the first cycle of chemotherapy. Apoptotic changes in 20% of CTCs [110]	After seven days of 5-FU, only 29.3% of patients were CTCs-positive compared to 80.5% before treatment [110]
PDAC patients [77]	Chemotherapy (modified FOLFIRINOX) [77]	Patients who received neoadjuvant chemotherapy had lower CTCs numbers across all identified populations [77]	High CTCs level was associated with early recurrence [77]
mBrC patients [66]	Chemotherapy + bevacizumab [66]	Significant reduction in CTCs counts in 16 out of 17 patients (94%) [66]	PFS 9.6 vs. 7.3 months (< 5 vs. ≥ 5 CTCs) OS NR vs. 23.1 months (< 5 vs. ≥ 5 CTCs) [66]
mBrC patients [66]	Chemotherapy + trastuzumab or lapatinib [66]	Significant reduction in CTCs counts in 9/9 patients (100%) [66]	PFS 14.5 vs. 16.1 months (< 5 vs. ≥ 5 CTCs) [66]
mBrC patients [66]	Chemotherapy + endocrine therapy [66]	Significant reduction in CTCs counts in 16/40 patients (35%) [66]	–
mBrC patients [66]	Aromatase inhibitor, Tamoxifen, Fulvestrant [66]	Significant reduction in CTCs counts in 1/10 patients (10%) [66]	PFS 14.1 vs. 3.5 months (< 5 vs. ≥ 5 CTCs) OS NR vs. 17.3 months (< 5 vs. ≥ 5 CTCs) [66]
mBrC patients [66]	Chemotherapy (various regimens) [66]	Significant reduction in CTCs counts in 15 out of 30 patients (50%) [66]	PFS 11.5 vs. 6.0 months (< 5 vs. ≥ 5 CTCs) OS 36.3 vs. 17.1 months (< 5 vs. ≥ 5 CTCs) [66]
Breast cancer patients [111]	Chemotherapy (SUCCESS trial protocol) [111]	435/2026 patients were CTCs-positive before chemotherapy (21.5%). 330/1493 patients were CTCs-positive after chemotherapy (22.1%) [111]	CTCs-positivity was associated with lymph node metastasis and predicted reduced DFS (88.1% vs. 93.7% after 36 months) [111]
Metastatic prostate cancer with AR-V7-positive CTCs [112]	Ipilimumab + nivolumab [112]	2/15 patients achieved a PSA response. 2/8 patients achieved an objective response. DRD + patients had longer PFS and OS than DRD- patients (p < 0.05) [112]	Ipilimumab plus nivolumab demonstrated encouraging efficacy in AR-V7-positive prostate cancers DNA-repair deficiency mutations but not in the overall study population [112]
In-vivo mouse tumor cell lines and NSCLC patients [83, 85]	Dual CD47 and PD-L1 blockade [83, 85]	Dose-dependent reduction of CD47 and PD-L1 expressions. Synergistic effect of anti-CD47 and anti-PD-L1 antibodies. It reduced tumor nodules in the lung without signs of treatment toxicity [83, 85]	The absence of PD-L1-positive CTCs predicts a sustained response to long-term immunotherapy. Isolated CTCs without quantified PD-L1 expression are unsuitable for identifying long-term survivors [83, 85]
In-vivo mice model with melanoma cell lines[94]	TA99 (anti-gp75) mAB [94]	Increased tumor cell phagocytosis. Control mAbs prolonged the macrophage-tumor cell contact. Increased number of CTCs in Kupffer cell-depleted mice compared to the control [94]	In the absence of mAbs, Kupffer cells sampled tumor cells but did not cause their elimination. Antibody-dependent phagocytosis efficiently removes CTCs in a murine tumor model [94]
In-vitro human blood samples; in-vivo mice model [98]	“Unnatural killer cell” therapy; E-Selectin /TRAIL [98]	ES/TRAIL functionalized leukocytes reduce the number of CTCs in the mouse bloodstream. ES-blocking antibodies inhibited the adhesion of ES/TRAIL liposomes to leukocytes. ES/TRAIL therapy effectively kills cancer cells in the presence of human blood [98]	ES/TRAIL liposomes functionalize leukocytes to kill cancer cells with negligible cytotoxicity to leukocytes and human endothelial cells. ES/TRAIL therapy effectively targets CTCs in human blood [98]
In-vitro; AML cell lines [113]	TRAIL/E-Selectin [113]	A dose-dependent decrease in all cell lines’ viability after TRAIL treatment. Therapy efficacy did not depend on the CD34 + status of the bone marrow cells. The exposure to TRAIL and E-selectin killed 30% of captured cells in 1 h [113]	The rolling delivery is more effective than static exposure to a TRAIL-immobilized surface. TRAIL treatment does not affect the functions of hematopoietic CD34 + stem cells [113]
Mice melanoma xenograft (B16F10) and triple-negative breast cancer tumor model [89, 114]	Platelet-aPD-L1 [89, 114]	aPD-L1 reduced cancer recurrence rate and prolonged mice OS. P-aPD-L1 was more efficient in therapy than free-aPD-L1 therapy. PD-L1 blockade induced a strong anticancer immune response [89, 114]	The concentrations of antibodies increase around cancer cells, while platelets release the conjugated aPD-L1 to recruit immune cells. Platelets effectively deliver drugs to CTCs and stop cancer spread [89, 114]
In-vitro mouse xenografts [115]	BYL719 (PIK3CA inhibitor), PD173074 (FGFR1 inhibitor) and AZD4547 (FGFR2 inhibitor) [115]	CTCs were highly sensitive to BYL719 and AZD4547 and moderately sensitive to PD173074. BYL719 with AZD4547 completely inhibited tumor growth in mouse xenografts [115]	The analysis of peripheral blood CTCs enabled the non-invasive detection of CTCs subclones to profile drug selectivity. PIK3CA and FGFR2 act synergistically [115]
In-vitro (CTC-MCC-41 cell line; metastatic CRC) [116]	MK2206 (AKT inhibitor) and RAD001 (mTOR inhibition) [116]	The PI3K/AKT/mTOR pathway inhibition and dose-dependent suppression of CTCs growth [116]	Dual inhibition of AKT and mTOR inhibits CRC growth in-vitro [116]
In-vitro (NCI-H460 cells); mice model [70]	Modified photodynamic + green fluorescent protein + photosensitizers [70]	The therapy was more effective in GFP + cells than in GFP- cells. Decreased CTCs count. Higher efficacy toward the GFP-positive cells. No changes in tumor size. Reduced lung metastasis and higher 1-week survival rate of treated mice [70]	The therapy directly eliminated CTCs and extended mice survival. CTCs are the core driver of distant metastasis. This approach is not suited for in-vivo targeting of CTCs [70]
TNBC xenografts [96]	ICAM1 [96]	ICAM1 overexpressed promotes spontaneous metastasis to the lungs. ICAM1 knockdown in MDA-MB-231 cells inhibited CTCs aggregation. After orthotopic implantation of MDA-MB-231 cells, anti-ICAM1 treatment reduced spontaneous lung metastasis but did not inhibit tumor growth [96]	ICAM1 may contribute to metastasis initiation in TNBC. High ICAM1 levels correlate with reduced metastasis-free survival in breast cancer. ICAM1 mediates CTCs aggregation, enhances tumor cell-endothelial cell crosstalk, and promotes tumor cell proliferation [96]
In-vitro; mouse model of breast cancer metastasis [117]	Urokinase-type plasminogen activator [117]	The number of CTCs clusters increased proportionally to the primary tumor growth. Urokinase-type plasminogen activator prevented the formation of CTCs clusters and prolonged mice survival [117]	In the bloodstream, CTCs clusters flow slower than single CTCs. The percentage of CTCs clusters increased in line with disease progression. The inhibition of CTCs clustering prevents early metastasis [117]
In-vitro; prostate cancer cells [103]	CAFs-targeted therapy [103]	“Reactive CAFs” and normal fibroblasts protect prostate tumor cells from shear stress and may promote cancer cell survival and metastasis [103]	“Reactive CAFs” support tumor survival and proliferation. Fibroblast-derived factors confer resistance to fluid shear stress [103]
CTCs derived from BrC (BR16 BRx50) [102]	Na + /K + ATPase inhibitors (Digitoxin and Ouabain) [102]	It reduced CTCs cluster size and dissociation. No effect on cell viability and proliferation. Methylation of genes related to stemness. Reduced ability of BR16 cells to survive during the early steps of metastasis [102]	Digitoxin and Ouabain dissociate CTCs clusters, reverse DNA methylation, and suppress metastasis. In CTCs clusters, genes related to stemness and metastasis are hypomethylated. Preventing CTCs cluster formation may hinder the spread of cancer [102]

If not stated otherwise, the units are [number of CTCs/7.5 ml blood]

*mAb* monoclonal antibody, *CTCs* circulating tumor cells, *mPrC* metastatic prostate cancer, *mBrC* metastatic breast cancer, *PDAC* pancreatic ductal adenocarcinoma, *HCC* hepatocellular carcinoma, *NSCLC* non-small cell lung cancer, *CRC* colorectal carcinoma, *OS* overall survival, *PFS* progression-free survival, *GFP* green fluorescent protein, *TNBC* triple-negative breast cancer, *CAF* cancer-associated fibroblasts, *DRD* DNA-repair deficiency, *P-aPD-L1* Platelet-anti-PDL

### Surgical resection

Surgical resection is the primary method of managing low-stage cancers. The excision of the primary tumor can also reduce the burden of circulating tumor cells and decrease the risk of early metastasis [71, 72]. However, we must address some discrepancies. While the primary tumor resection decreased CTC’s volume in an orthotopic HCC model, the same intervention in HCC patients had the opposite effect [71, 73]. The increase in CTCs volume appeared to depend on the presence of CTCs macroscopic tumor thrombi. During liver rotation [74], the HCC cell was forced into the bloodstream from the hepatic vein tumor thrombi and caused cancer spread. In desmoplastic cancers, such as pancreatic ductal adenocarcinoma (PDAC), stroma often forms enclosed compartments, potentially slowing tumor progression and preventing early metastasis [75]. Its removal during surgery may paradoxically facilitate cancer spread. However, Tamminga et al. did not observe increased CTCs volume after lung cancer surgery [76].

### Chemotherapy

Chemotherapy remains the first-line treatment in multiple advanced cancers. This approach is usually chosen considering the primary tumor stage, and the effect on CTCs count seems secondary to the systemic toxicity of used drugs. Chemotherapeutics effectively reduce the number of CTCs and decrease the risk of early metastasis [77]. In metastatic breast cancer, chemotherapy alone reduced the count of CTCs in the blood of 15 out of 30 patients (50%). Patients with low CTCs had significantly longer progression-free survival and overall survival than patients with higher CTCs volume [78]. Nevertheless, the lack of target-specificity hinders the introduction of CTC-oriented therapy [79]. A more selective approach is required since CTCs differ genetically and phenotypically from primary tumor cells.

Interestingly, CTCs showed significant heterogeneity even within the same patient. Some acquired hybrid epithelial-mesenchymal phenotype, as if during a partial endothelial-mesenchymal transformation. They became more sensitive to tumor environmental stimuli and gained the ability to colonize distant organs [25, 80]. Mesenchymal-like cells in the bloodstream are associated with progressive disease post-therapy in breast cancer patients. Chemotherapy may induce a CTCs phenotype switch by enforcing the selective survival of resistant clones and increasing the number of CTCs with mesenchymal features [80–82]. Since cancer stem cells and partial-EMT CTCs are resistant to conventional chemotherapy, the increase in their number predicts a lack of long-term efficacy and a worse prognosis.

### Immune checkpoint blockade

CTCs and distant metastasis cells have different phenotypes than the primary tumor. They may resist first-line therapy—most express surface proteins, such as PD-L1 and CD47, facilitate immune surveillance escape [83]. The interaction between those proteins and their respective ligands on the surface of immune cells causes T-cell suppression and impairs antitumor response [84]. In-vivo, the absence of PD-L1-positive CTCs can predict sustained response to long-term immunotherapy [85]. Therefore, PD-L1-expressing CTCs became a potential therapeutic target [86].

Dual immune checkpoint blockade may further enhance the efficacy of therapy. The co-inhibition of CD47 and PD-L1 decreased lung cancer nodules in mice dose-dependently [83]. However, selecting a therapeutic group is usually complex and may require real-time assessment of biomarker dynamics depending on the type of disease. The analysis of CTCs from patients’ peripheral blood emerges as a non-invasive tool to determine the indications for immunotherapy and predict the resistance to therapy [87]. Clinical trials evaluating the changes in the count and phenotype of CTCs during immunotherapy may determine whether CTCs can be implemented into clinical practice [88].

### Platelets-targeted therapy

In the bloodstream, CTCs associate with platelets and form clusters. Those complexes can withstand shear force, limit antigen presentation on the surface of tumor cells, and evade the immune response [89, 90]. Platelet coating protects CTCs from T and natural killer cells, facilitating their spread and early metastasis [65, 91].

Interestingly, platelets adhere to the injury side upon vascular endothelial cells’ damage and release their contents, invoking an immune response and forming platelet-derived microparticles (PMP) [92]. PMPs promote the binding of anti-PD-L1 to CTCs, block PD-L1 on tumors and antigen-presenting cells (APCs), and inhibit metastasis [92, 93]. In mice with TNBC and primary melanoma, this approach effectively released aPDL-1 during platelet activation, reducing the risk of cancer recurrence and prolonging mice survival. Since the concentration of antibodies increases around cancer cells, Platelets-aPDL-1 conjugates are more effective than free anti-PD-L1 therapy. Furthermore, platelet activation recruits other immune cells, which, after the PD-L1 blockade, can induce a strong anticancer immune response [89].

### Monoclonal antibodies

Recently, monoclonal antibodies have become a frontline strategy to treat cancer. However, their role in targeting CTCs still needs to be defined. Antibody-dependent phagocytosis by murine Kupffer cells can remove CTCs from the bloodstream [94]. This effect depends on FcγRI and FcγRIV, which are required to prevent liver metastasis [95].

ICAM1 overexpression in lung and breast cancer promotes spontaneous metastasis to the lung and is associated with shorter survival in breast cancer [96]. ICAM1 levels are higher in CTCs clusters than in single CTCs, increase upon clustering, and enhance cancer stemness and cell-cycle progression. In the orthotopic model, anti-ICAM antibodies inhibited CTCs aggregation and reduced spontaneous lung metastasis but did not impact the primary tumor growth [96]. Although data regarding monoclonal antibodies targeting CTCs is limited, the results prompt further investigation.

### Immunomodulation

Cancer cells associate with platelets during the migration in the bloodstream and release immunosuppressive factors, such as cytokines, cell surface proteins, and growth factors, to avoid the immune response. Under their influence, tumor-associated cells suppress the ability of immune-competent cells to present antigens and eliminate tumor cells. Therefore, methods that enhance the ability of immune cells to recognize and execute CTCs have been proposed.

CTCs must interact with white blood cells and cross vascular endothelial cells before they can extravasate. In many CTCs, surface-expressed ligands bind to E-selectin (ES) expressed on endothelial cells, which triggers the death receptor TRAIL-induced autophagy of tumor cells [97]. Furthermore, white blood cells carrying ES and TRAIL liposomes can directly promote CTCs phagocytosis, reduce CTCs count, and prevent metastasis [98].

Cancer-associated fibroblasts (CAF) are populations of fibroblasts that acquire immunomodulatory properties through the crosstalk with tumor cells, facilitate immune escape, and drive tumor progression [99]. CAFs circulate in the bloodstream, form complexes with CTCs, and protect them from an anticancer immune response. The number of circulating cCAFs/CTCs clusters increases in the blood of advanced breast cancer patients and is associated with poor prognosis. In-vitro, cCAFs/CTCs clusters were present only in metastatic breast cancer, while no such phenomenon occurs in cancers without metastasis [100]. Therefore, disrupting the formation of cCAF/CTCs complexes may be a potential therapeutic strategy.

While many researchers focus on reprogramming CAFs [101], the therapeutic potential of targeting CTCs and their clusters still needs to be explored. Prolonged treatment with ouabain and digitoxin, Na + /K + ATPase inhibitors, caused CTCs cluster dissociation and preserved optimal proliferation compared to control cells without causing generalized DNA methylation [102]. It also reduced the ability of BR16 cells to survive during the early steps of metastasis, hindering cancer spread. CAFs and prostate cancer cells form conglomerates that enhance CTCs’ survival in fluid shear stress [103, 104]. CTCs cluster integrity corresponds with disease progression and is more compact in a more advanced stage. Thus, CAFs dissociation may limit prostate cancer metastasis and increase therapy efficacy [105–107].

## CTCs and tumor-associated macrophages

Tumor-associated macrophages (TAMs) are immune cells that reside within the tumor microenvironment and promote tumor dissemination via direct contact with tumor cells [118]. TAMs secrete growth factors, cytokines, and chemokines, supporting EMT and cancer cell proliferation migration. They enhance proliferation by secreting growth factors, cytokines, and chemokines [118, 119]. They also degrade the extracellular matrix, promoting the migration and CTCs attachment to vascular endothelium. The crosstalk between TAMs and CTCs seems crucial for forming distant metastasis [120, 121]. Hence, targeting TAMs has emerged as a strategy to reduce metastases [118].

### The emerging role of TAMs in CTCs-targeted therapies

Macrophages play a crucial role in tissue repair and defend the organism from pathogens. Distinct populations of macrophages play different roles in the immune response. However, their functions are not set in stone and may change depending on the microenvironmental cues [122]. In cancer, their proinflammatory activity may drive tumorigenesis and metastasis [123].

**Fig. 2:**
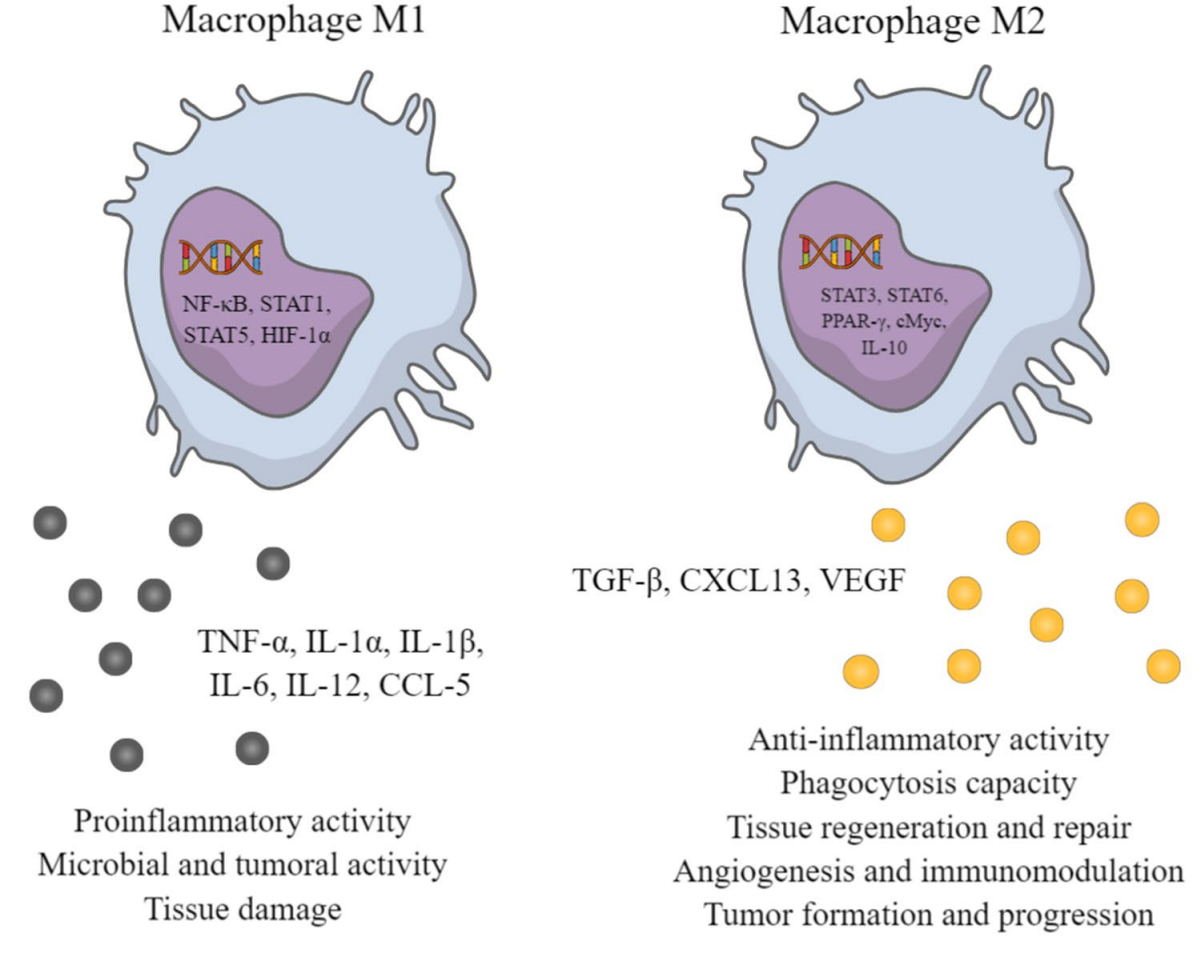
The function of macrophages’ subtypes. Macrophages can be divided into two main subtypes. The antitumor M1 macrophages release proinflammatory cytokines, facilitating proinflammatory, microbial, and tumoral activity. It is widely responsible for tissue damage. The immunosuppressive M2 macrophages facilitate tumor growth by secreting anti-inflammatory cytokines. They also present phagocytosis capacity and anti-inflammatory activity. M2 macrophages are involved in tissue regeneration, repair, angiogenesis, immunomodulation, and tumor formation and progression. *TNF-α* tumor necrosis factor α, *IL* interleukin, *CXCL* chemokine C-X-C motif ligand, *CCL* chemokine C–C motif ligand, *TGF-β* transforming growth factor β, *VEGF* vascular endothelial growth factor, *NF-κB* nuclear factor kappa-light-chain-enhancer of activated B cells, *STAT* signal transducer and activator of transcription, *HIF* hypoxia-inducible factor, *PPAR* peroxisome proliferator-activated receptor

TAMs are a population of macrophages that infiltrate tumors and contribute to the development and progression of cancer. TAMs can be classified into two major subtypes: proinflammatory M1 macrophages—which exhibit antitumor activity by secreting interleukin-12 (IL-12), tumor necrosis factor-alpha (TNF-α), and interferon-gamma (IFN-γ) [124]—and immunosuppressive M2 macrophages, promoting tumor growth via the secretion of interleukin-10 (IL-10) and transforming growth factor-beta (TGF-β) (Fig. 2) [125, 126].

TAMs in the tumor microenvironment are associated with poor clinical outcomes in various cancers, including breast, ovarian, and lung cancer [127]. They stimulate angiogenesis, suppress antitumor immune responses, and remodel the extracellular matrix [128]. In this way, TAMs favor the development of an immunosuppressive niche in which cancer cells undergo EMT, acquire resistance to apoptosis, and proliferate. Therefore, targeting TAMs has emerged as a promising approach to improve the efficacy of existing treatments [123]. Recent studies have provided new insight into cancer biology, which raised the inhibition of TAMs recruitment to the tumor site, their repolarization to an antitumor phenotype, or depletion to the forefront of macrophage-targeting therapies [123, 129].

### The ominous crosstalk between TAMs and CTCs

**Fig. 3:**
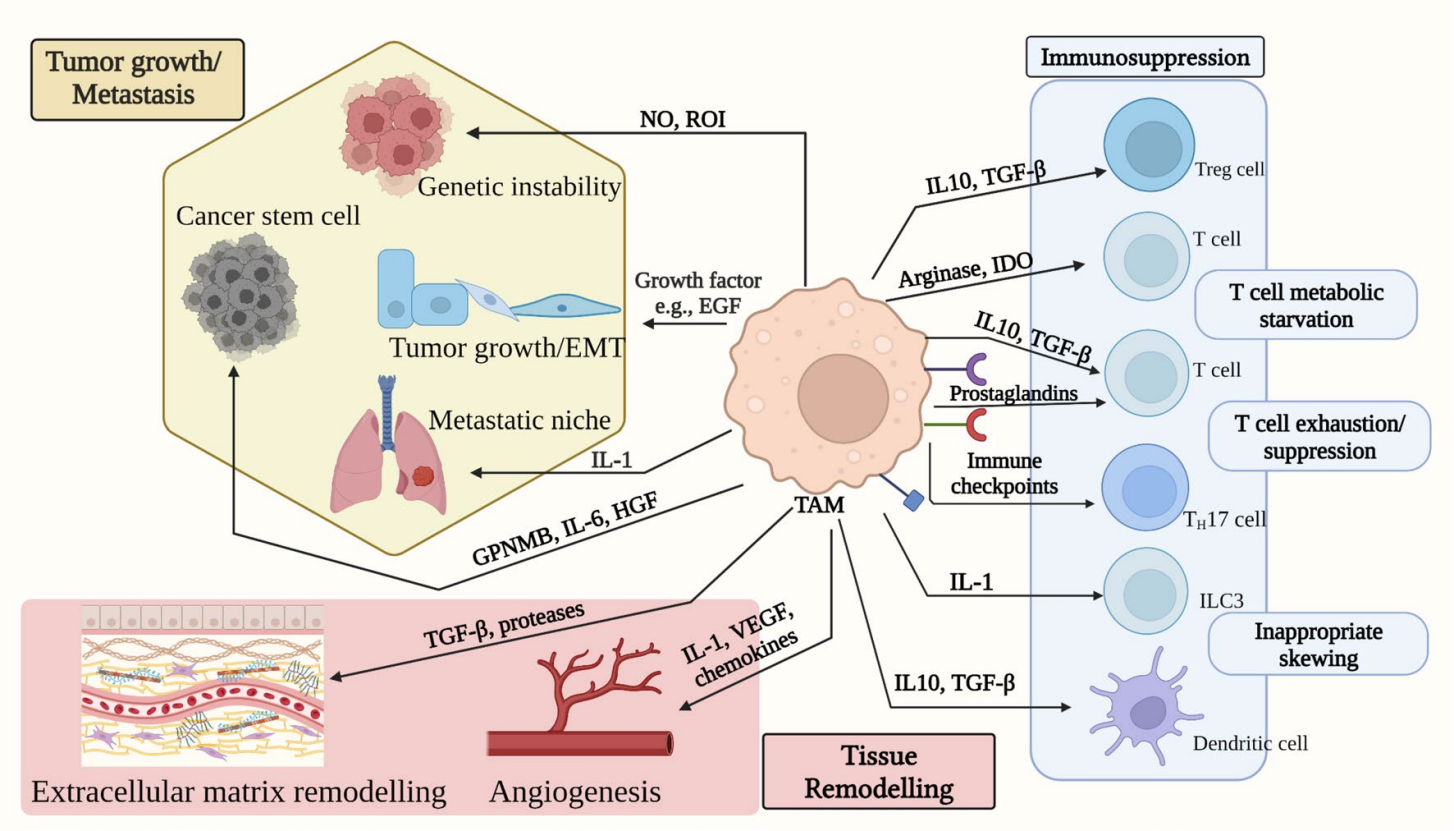
Tumor-associated macrophages (TAMs) and their implications for metastasis. TAMs participate in various stages of tumorigenesis. TAMs produce NO and reactive oxygen intermediates during cancer initiation, which induce DNA damage and genetic instability. TAM-derived EGF, VEGF, HGF, IL-6, and GPNMB promote cancer stem cell proliferation. TAMs also secrete IL-1 and TGF-β, which are involved in ECM remodeling and cancer dissemination. IL-10, TGF, prostaglandins, and IDO stimulate regulatory T cell growth, dendritic cells’ immune tolerance, and T cell metabolic deprivation. Immunosuppressive TAMs exhibit increased expression of immune-checkpoint markers (PD-L1, PD-L2, B7-H4), contributing to T cell exhaustion. *ILC3* type 3 innate lymphoid cell, *Th17* T helper 17, *Treg* T regulatory, *EMT* epithelial-mesenchymal transition, *NO* nitric oxide, *ROI* reactive oxygen intermediates, *GPNMB* glycoprotein non-metastatic b, *IL* interleukin, *EGF* epidermal growth factor, *HGF* hepatocyte growth factor, *VEGF* vascular endothelial growth factor, *TGF-β* tumor growth factor-β, *IDO* indoleamine 2,3-dioxygenase, *ECM* extracellular matrix. “Created with BioRender.com.”

Macrophages can interact with CTCs through cell-to-cell contact or the secretion of growth factors and cytokines (Fig. 3). On the one hand, TAMs secrete vascular endothelial growth factor or fibroblast growth factor—and cytokines (i.e., IL-6, TNF-α, and TGF-β) to promote CTCs survival, proliferation, and migration [126, 130].

On the other hand, macrophages express cell surface receptors that interact with ligands expressed by CTCs. For instance, CD47, a receptor expressed by CTCs, interacts with the macrophage surface receptor signal regulatory protein alpha (SIRPα) [131]. Their interaction inhibits macrophage phagocytosis of CTCs and allows CTCs to evade immune surveillance. Blocking the CD47-SIRPα interaction can promote macrophage-mediated phagocytosis of CTCs and improve antitumor immunity [132]. The interactions between CTCs, macrophage integrins, and toll-like receptors (TLRs) promote CTCs adhesion, migration, and invasion [133, 134]. Since TLR4 promotes CTCs migration in pancreatic cancer and blocking TLR4 signaling inhibits CTCs migration and invasion, TLR4 appears to be a potential therapeutic target [135].

Macrophages can also transfer exosomes to CTCs. Exosomes are small, cell-derived vesicles that move proteins, lipids, and nucleic acids between cells [134]. Macrophage-derived exosomes transfer growth factors and cytokines, promoting CTCs proliferation, migration, and invasion [136]. Tumor cell-derived exosomes can switch macrophage phenotype to M2 and suppress antitumor immunity [137].

Interestingly, macrophages can also induce CTCs apoptosis and inhibit CTCs proliferation. Activated M1 macrophages produce reactive oxygen and nitrogen species, causing DNA damage, and secrete TRAIL and IFN-γ to induce apoptosis in CTCs [138, 139]. Moreover, macrophages can directly eliminate CTCs via phagocytose [140].

### Macrophage-targeting therapies

Since macrophages have emerged as a promising therapeutic target, reports discussing the rationale of inhibiting the activity of M2 macrophages, promoting M2 macrophage differentiation, or modifying the functions of a given macrophage population started appearing. In the following section, we will shortly review the available methods that target TAMs and can soon complement CTCs-targeting approaches.

#### Macrophage-targeting agents

Macrophage-targeting agents, such as bisphosphonates, liposomes, and nanoparticles, can exploit the ability of macrophages to phagocytose foreign particles [141] and express specific surface receptors [142]. Bisphosphonates, commonly used to treat bone metastases, inhibit the activity of M2-like macrophages and reduce tumor growth and metastasis in preclinical models [143].

#### Immunotherapeutic agents

Immunotherapeutics can modulate macrophage polarization. IL-12 and IFN-α switch toward the M1 phenotype and enhance antitumor immunity [144, 145]. Blocking signaling pathways that promote M2-like macrophage polarization, such as the TGF-β pathway, enhances antitumor immunity and improves therapeutic outcomes in preclinical models [125].

Monoclonal antibodies against macrophage surface markers, such as CD40, CD47, and CD163, induce macrophage activation and antitumor immune responses [146, 147]. Immunomodulatory drugs, such as lenalidomide and thalidomide, inhibit the secretion of pro-tumor cytokines by M2-like macrophages and stimulate the secretion of antitumor cytokines by M1-like macrophages [148, 149].

#### Gene therapy

Gene therapy involves the introduction of genes encoding molecules that can modify the behavior of macrophages within the tumor microenvironment [122, 150]. For instance, reprogramming macrophages to switch from the tumor-promoting M2 phenotype to the tumor-inhibiting M1 phenotype can be achieved by using viral vectors, which deliver genes encoding cytokines or chemokines activating the M1 phenotype, such as IFN-γ and IL-12 [122, 150].

Gene therapy can also deliver genes encoding molecules that target specific signaling pathways or molecules involved in macrophage-mediated tumor progression. Macrophages transfected with small hairpin RNA (shRNA) targeting the C–C motif chemokine receptor 2 (CCR2) via a lentiviral vector reduced macrophage infiltration and tumor growth in a preclinical model of breast cancer [151].

In addition, non-viral methods such as electroporation, liposome-mediated transfection, and CRISPR-Cas9 gene editing have also been explored for gene therapy approaches in targeting macrophages in cancer [152].

#### Tumor microenvironment modulation

Several studies have also explored using drugs that modulate the tumor microenvironment to reprogram macrophages. Vascular disrupting agents, such as combretastatin A4 phosphate (CA4P), selectively induce tumor hypoxia, leading to the recruitment of M1-like macrophages, suppression of M2-like macrophages [153], and enhancing antitumor immune response [154]. Chemotherapeutics, such as gemcitabine, also favor the switch toward the M1 phenotype in the tumor microenvironment [155].

#### Extracellular vesicles

Extracellular vesicles (EVs) are nanosized lipid bilayer structures released by cells into the extracellular environment. They play a crucial role in cell-to-cell communication and carry a variety of biomolecules, such as proteins, lipids, and nucleic acids. EVs can cross biological barriers, including the blood–brain barrier, and cells selectively and efficiently deliver therapeutic cargo to target [156]. Therefore, they have emerged as attractive carriers of therapeutic agents.

Several studies have explored the potential of EVs as delivery vehicles for targeting macrophages in cancer therapy. For instance, EVs can be engineered to express specific ligands or antibodies to target macrophage surface markers and modify their functions selectively [156]. EVs can also be loaded with particular cargo, such as siRNA or miRNA, that can modulate macrophage polarization towards an antitumor phenotype. Moreover, EVs derived from mesenchymal stem cells can target and modulate the function of macrophages within the tumor microenvironment, enhancing antitumor immune response [157].

## Conclusion

The last years have brought immense progress in detecting and targeting circulating tumor cells. CTCs detected in patients’ bloodstream are associated with more advanced diseases and predict poor prognosis in multiple cancers. EpCAM, vimentin, CD44, and TGM2 have emerged as promising CTCs biomarkers, which study allows for determining molecular characteristics of CTCs. Dynamic analysis of CTCs and molecular markers on their surface, such as PD-L1, can predict clinical response to immunotherapy, help monitor disease course, and guide personalized therapy. However, the heterogeneity and constant evolution of CTCs phenotype make their analysis challenging. Further research is required to utilize their role in cancer therapy fully.

Targeting CTCs has emerged as a promising therapeutic strategy to improve cancer treatment outcomes. By eliminating or preventing the spread of CTCs, clinicians aim to reduce the risk of metastasis and prolong patients’ survival. Reducing the CTCs count via surgical resection, chemotherapy, and immunotherapy can reduce the risk of progression, but those methods have inherent limitations. CTCs localized in venous thrombi can be easily spread due to careless handling during the procedure; the “no touch” approach may limit the number of CTCs.

Chemotherapy and immunotherapy are associated with systemic toxicities, which may diminish their beneficial effects on CTCs volume. Platelet coating and interactions with blood cells in the bloodstream impart CTCs’ antigen presentation and improve their adhesive properties, facilitating immune surveillance escape and CTCs anchoring to the vascular endothelium at the metastatic site. Both mechanisms diminish the efficacy of systemic therapy and may require targeted therapy, but such approaches have yet to be tested in clinical settings.

CTCs extensively interact with TAMs through cell-to-cell contact or by secreting soluble factors that attract TAMs to the tumor site. TAMs promote CTCs’ survival and dissemination by secreting growth factors and cytokines. They also contribute to forming a supportive environment for CTCs by remodeling the extracellular matrix and promoting angiogenesis. TAM-targeted therapies aim to decimate the population of tumor-suppressing M2 macrophages, enhance the antitumor activity of M1 macrophages, or force an M2-to-M1 phenotype switch to interrupt the positive feedback loop between TAMs and CTCs.

Since CTCs significantly impact patients’ prognosis, their detection is essential for accurate diagnosis and personalized therapy. Targeting CTCs holds on the premise of improving patient outcomes. Biomarkers such as EpCAM, CD44, and TGM2 improve the reliability of CTCs detection, but the constantly evolving phenotype of CTCs may limit their utility. Further research into the detection, interactions, and evolution of CTCs may improve our understanding of their role in cancer metastasis and increase the accuracy of patient prognosis.

## Data Availability

The data presented in this study are available on request from the corresponding author.
